# PACAP and Maxadilan (PAC1 Agonist) Influence Plaque Progression, Migratory Ability, and Mitochondrial Morphology and Dynamics in Vascular Smooth Muscle Cells

**DOI:** 10.3390/cells15121127

**Published:** 2026-06-22

**Authors:** Julia Brauschke, Lisa-Marie Schütz, Gabriel A. Bonaterra, Ralf Kinscherf, Anja Schwarz

**Affiliations:** 1 Department of Medical Cell Biology, Institute of Anatomy and Cell Biology, Philipps-University of Marburg, 35037 Marburg, Germany; brausch4@students.uni-marburg.de (J.B.); gabriel.bonaterra@staff.uni-marburg.de (G.A.B.); ralf.kinscherf@staff.uni-marburg.de (R.K.); 2 Department of Public Health and Environmental Health, Apollon University of Applied Sciences, 28359 Bremen, Germany; marie.lisa.schuetz@gmail.com

**Keywords:** PACAP, Maxadilan, oxLDL, atherosclerosis, HCASMC, apoptosis, migration, mitochondria

## Abstract

**Highlights:**

**What are the main findings?**

- PACAP deficiency in vivo increased lumen stenosis but reduced plaque burden in atherosclerotic mice, while in vitro PACAP enhanced the viability of oxLDL-treated human coronary artery smooth muscle cells (HCASMCs).
- Maxadilan, a PAC1 agonist, improved migration in oxLDL-impaired HCASMCs, and both oxLDL and PACAP influenced mitochondrial morphology.

**What are the implications of the main findings?**

- The findings highlight the complex role of the PACAP/PAC1 system in vascular pathology, suggesting that its targeted modulation could offer novel strategies for stabilizing atherosclerotic plaques.
- Specifically, activating PAC1 or supplementing PACAP may help balance plaque formation and vascular function, warranting further investigation for therapeutic applications.

**Abstract:**

Background: Pituitary adenylate cyclase-activating polypeptide (PACAP) functions as an anti-atherogenic neuropeptide. Maxadilan, a PAC1 receptor agonist, offers atheroprotection by acting downstream of vascular inflammation caused by hypercholesterolemia. This study aims to explore how PACAP and Maxadilan influence migration and apoptosis in human coronary artery smooth muscle cells (HCASMCs). Methods: To investigate the role of PACAP deficiency in the pathogenesis of atherosclerosis under standard chow (SC) in vivo, PACAP^−/−^-mice were crossed with ApoE^−/−^-mice to generate PACAP^−/−^/ApoE^−/−^-mice. The whole aorta was isolated and stained with OilRedO (ORO). Atherosclerotic lesions and lumen stenosis in the brachiocephalic trunk were quantified using ImageJ 1.54p (Fiji). To further investigate the role of PACAP and Maxadilan in the pathogenesis of atherosclerosis with special respect to HCASMC under a lipid-enriched environment, HCASMCs were treated with oxLDL, with or without PACAP or Maxadilan. Uptake and accumulation of oxLDL were analyzed using Bodipy^TM^493/503, and cell viability was assessed with PrestoBlue^®^. Cell migration was evaluated using the scratch assay and the MRI wound-healing tool in ImageJ (Fiji). Mitochondrial morphology was examined with MitoTracker Green and the MiNA tool in ImageJ (Fiji). Apoptotic processes were analyzed by Western blot, immunocytofluorescence staining, and ELISA. Results: In vivo, PACAP^−/−^/ApoE^−/−^-mice showed increased lumen stenosis and decreased plaque burden compared with ApoE^−/−^-mice. In vitro, PACAP enhanced the viability of oxLDL-treated HCASMCs, while neither PACAP nor Maxadilan influenced lipid content in HCASMCs, regardless of oxLDL presence. Both oxLDL and PACAP slowed cell migration, but Maxadilan increased migration in oxLDL-treated HCASMCs. The protein level of the proliferation marker Ki67 was reduced in cells treated with oxLDL and Maxadilan. Additionally, BAX, which promotes intrinsic apoptosis, was elevated in HCASMCs stimulated with Maxadilan and oxLDL. Investigations of mitochondrial morphology indicated that oxLDL and PACAP increased the individual and network structures, with a decrease in branches per network. Conclusion: Our data highlight the complex role of the PACAP/PAC1 system in vascular pathology and suggest that selective modulation—such as targeted PAC1 activation or PACAP supplementation—could lead to new strategies for stabilizing atherosclerotic plaques. In the long term, this could improve the balance between plaque formation and vascular function.

## 1. Introduction

Cardiovascular diseases [CVDs] are the leading cause of death worldwide. In 2019, an estimated 17.9 million people died from CVDs, accounting for 32% of all deaths globally, with 85% due to heart attacks and strokes [1]. Atherosclerosis, the main cause of coronary artery disease, is a chronic inflammatory condition caused by endothelial dysfunction and exacerbated by oxidative stress and the accumulation of lipids in the arterial wall, as well as by necrosis, fibrosis, and calcification [2].

Low-density lipoprotein (LDL), particularly in its modified forms, is the primary factor contributing to lipid accumulation in atherosclerotic lesions. Modifications of LDL, such as oxidation with subsequent scavenger receptor-mediated internalization by cells, e.g., macrophages (MΦs) and smooth muscle cells (SMCs), stimulate foam cell formation and trigger an immune response, resulting in circulating low-density lipid (LDL)-containing immune complexes that are highly atherogenic. The stability of atherosclerotic plaques depends on the thickness of their fibrous cap and the level of local inflammation. Cap thinning, driven by the death of vascular SMCs (VSMCs), as well as collagen and extracellular matrix (ECM) breakdown, increases the risk of plaque rupture, leading to myocardial infarction, angina, or stroke [3].

VSMCs occur and participate in all phases of atherosclerosis, from early lesions to advanced plaques [4]. These cells are fusiform, exhibit a low proliferation rate, and express specific contractile proteins such as smooth muscle myosin heavy chain, SM22, and Calponin [5,6]. The plasticity of VSMCs plays a crucial role in vascular diseases, including the development and progression of atherosclerosis [7,8]. Initially, VSMCs switch from a contractile (differentiated) to a synthetic S-phase (dedifferentiated phenotype), losing typical markers [9,10,11,12,13]. As they lose their contractile phenotype in atherosclerosis, they start to express genes associated with other cell phenotypes [9,10,11,12]. VSMCs can differentiate into osteoblast- or chondrocyte-like cells (Runx2, Msx2, Fn1, Col1a1, Col1a2), macrophage-like cells (CD68, Lgals3), mesenchymal-like cells (Sca1, Eng), endothelial-like cells (Vcam1), or myofibroblasts (Pdgfßr) [10,11,12]. In human and mouse atherosclerosis, VSMCs make up at least 50% of foam cells, express markers of diverse cell types, such as CD68, and lose α-SMA expression [14,15]. While VSMC proliferation can be advantageous during atherogenesis, VSMC apoptosis, senescence, and the formation of macrophage-like cells from VSMCs may enhance inflammation within the plaque [3].

In 1989, PACAP, a neuropeptide, was isolated from ovine hypothalamic extracts and identified in two forms: PACAP27 and PACAP38 [16]. PACAP influences many biological functions by binding to specific G protein-coupled receptors on the plasma membrane, which are classified into subfamily II. These include (1) the VPAC1 and VPAC2 receptors, which bind VIP and PACAP with equal affinity (Kd = 1.0 nM), and (2) the PAC1 receptor, which is selective for PACAP (Kd = 0.5 nM) [17,18,19,20]. Our previous research shows that PACAP deficiency accelerates the formation and growth of atherosclerotic plaques in ApoE^−/−^-mice after 30 weeks on a standard chow (SC), by promoting inflammatory factors, autophagy, apoptosis, necroptosis, and fibrosis [21]. We also found that PACAP plays an essential role in regulating plaque inflammation via VPAC1 signaling and influences lipid homeostasis across different MΦ subtypes by modulating foam cell development [22]. Additionally, PAC1 deficiency increased markers of apoptosis, autophagy, necroptosis, and inflammation in plaques of ApoE^−/−^-mice after 30 weeks on an SC. Interestingly, PAC1 deficiency reduced luminal stenosis caused by a cholesterol-rich diet in ApoE^−/−^-mice, without altering hyperlipidemia [23]. Consequently, PACAP is considered a protective neuropeptide against atherosclerosis [21], while PAC1 deficiency appears to slow disease progression in ApoE^−/−^-mice [23]. Maxadilan, a PAC1 receptor agonist derived from sandflies [24], exhibits anti-atherogenic effects when injected into ApoE^−/−^-mice, likely due to its anti-apoptotic activity in VSMCs [25]. Furthermore, PACAP and PAC1 are expressed in chondroid cell cultures, where PACAP regulates cartilage matrix production and is involved in extravascular cartilage formation and osteogenesis [26,27]. Recently, it has been demonstrated that the absence of PAC1 in atherosclerotic lesions of ApoE^−/−^-mice has an anti-chondrogenic effect, likely by altering the extracellular matrix and shifting VSMCs toward a chondrocyte-like phenotype [28]. In this context, this study examined the roles of PACAP and Maxadilan in VSMC behavior concerning lipid homeostasis, viability, and migration, and their impact on mitochondrial function following oxLDL-induced cell stress.

## 2. Materials and Methods

### 2.1. Animals

To generate PACAP knockout mice (PACAP^−/−^), PACAP was deleted from the PACAP gene locus in C57BL/6 mice [29]. Then, PACAP knockout mice (PACAP^−/−^) were crossbred with ApoE^−/−^ (Charles River, Sulzfeld, Germany) to generate PACAP^−/−^/ApoE^−/−^ mice [21]. For this study, only male homozygous PACAP^−/−^/ApoE^−/−^ and ApoE^−/−^ mice were used, which were fed with SC for 30 weeks (LASQCdiet^®^ Rod16 Rad; LASvendi, Soest, Germany). All animals had ad libitum access to water and feed in their cages, which had a minimum area of 100 cm^2^ and an adequate enrichment device. The procedures complied with the regulations for animal experiments at Philipps-University Marburg.

### 2.2. Genotyping

Genomic DNA was isolated from the mouse ear using a commercial kit (DNA Extraction Solution; Peqlab, VWR Company, Erlangen, Germany) according to the manufacturer’s instructions (DirectPCR^®^ lysis reagent ear; Peqlab, VWR International; Darmstadt, Germany). Homozygous transgenic mice were subsequently identified by polymerase chain reaction (PCR) using intron-spanning oligonucleotides [21,22,29].

### 2.3. Dissection and Tissue Harvesting

At the age of 30 weeks, the mice were weighed, narcotized, and analgized with a combination of ketamine (150 mg/kg) and xylazine (20 mg/kg). Body size was determined by measuring nasal-to-anal length, and body mass index (BMI) was calculated as the ratio between body weight and surface area (g/cm^2^) [30]. The body surface area was derived from the DuBois equation: body surface (m^2^) = 0.007184 × weight [kg]^0.425^ × body size [cm]^0.725^ [30]. Immediately, after opening the thoracic cavity and the right atrium, heparinized (0.25 I.U./mL, Roche) blood samples were collected. Plasma was obtained by centrifugation (10 min, 650× *g*) and stored at −80 °C. The whole aorta and its branches, including the brachiocephalic trunk (BT), carotid, subclavian, renal, and common iliac arteries, were excised under direct observation through a binocular loupe fixed in 4% formaldehyde in Dulbecco’s phosphate-buffered saline (DPBS) for 24 h [31]. For the investigation of lumen stenosis, the BT was harvested using a binocular loupe, embedded in Tissue-Tek^®^ (Sakura Finetek, Staufen, Germany), and frozen in liquid nitrogen-cooled isopentane.

### 2.4. OilRedO Staining

The aorta was stained for 90 min at room temperature (RT) with fresh OilRedO (Sigma-Aldrich Chemie GmbH, Taufkirchen, Germany) solution (0.5% ORO in methanol + 1 M NaOH), then washed with 78% methanol at RT and rinsed three times with ddH_2_O. OilRedO stains lipid-rich plaques red, while areas without plaques remain pale. The carotid, subclavian, renal, and iliac arteries were removed. The aorta was dissected longitudinally from the ascending aorta to the origins of the renal arteries. The morphometric analysis was performed using ImageJ 1.54p (Fiji) software.

### 2.5. Immunohistology

Cryosection series (6 µm) of the BT were prepared for morphometric studies. The extent of atherosclerotic plaques in the BT was measured by computerized morphometry. These images were analyzed and quantified using ImageJ 1.54p (Fiji software) [32]. For this purpose, standard hematoxylin–eosin (HE) staining was performed. The lumen stenosis was determined by recording the lumen and plaque areas along the internal elastic lamina (or luminal plaque circumference) and calculating [[plaque area (µm^2^)]/[lumen area (µm^2^)] × 100% = lumen stenosis (%)] [21]. Media thickness was determined by measuring the area of the lumen along the internal elastic lamina and the area along the outer elastic lamina, and then calculating [lumen area to outer elastic lamina (µm^2^)]-[lumen area to internal elastic lamina (µm^2^)] = area of media (µm^2^)]. Quantification of immunoreactive plaque area was assessed as described previously [22,33].

### 2.6. Cell Culture

Human coronary artery smooth muscle cells (HCASMCs; Provitro AG, Berlin, Germany) were cultured in smooth muscle cell growth medium (Provitro AG) supplemented with a smooth muscle supplement mix (Provitro AG, Berlin, Germany). Cells were maintained at 37 °C in a 5% CO_2_ atmosphere, with the medium replaced every 2–3 days. All experiments utilized cells at passage 9 or lower. HCASMCs were treated for maximal 24 h with 0.1, 0.5, or 1.0 nM PACAP38 (Bachem AG, Bubendorf, Switzerland) and 0.1, 0.5, or 1.0 nM Maxadilan (Bachem AG, Bubendorf, Switzerland), followed by 25 µg/mL oxidized (ox)LDL.

### 2.7. LDL Oxidation

Oxidation of native (n) LDL (RayBiotech Inc., Peachtree Corners, GA, USA) was performed as described by Galle and Wanner [34] and Steinbrecher [35]. The nLDL was suspended in endotoxin-free phosphate-buffered saline (PBS) without Ca^2+^ and Mg^2+^ (LONZA, Ratingen, Germany) to a final concentration of 1 mg protein/mL and dialyzed using the Vivaspin™ 20-System (Thermo Fisher Scientific GmbH, Schwerte, Germany). The Vivaspin™ 20 centrifugal concentrator was sterilized with 70% ethanol for 10 min at 3000× *g*, then washed with endotoxin-free distilled water. The nLDL suspension in PBS was transferred into the Vivaspin™ 20 and centrifuged for 20 min at 4500× *g*. Two washing steps with PBS removed EDTA from the nLDL. OxLDL was obtained by incubating nLDL with 5 μM CuSO_4_ in calcium- and magnesium-free PBS at room temperature for 24 h. The oxidation level was validated using three methods: (1) 2,4,6-trinitrobenzene sulfonic acid (TNBSA) assay (Thermo Fisher Scientific Inc.) to detect and quantify free amino groups in peptides and proteins [36], (2) relative electrophoretic mobility (REM) via agarose gel electrophoresis and Coomassie Blue staining [37], and (3) spectrophotometry measuring absorbance spectra between 400 and 700 nm [34]. OxLDL showed an 11% ± 5% increase in REM, a 41.6% ± 7.8% increase in blocked amino groups compared to nLDL, and a loss of the characteristic absorption peaks at 460 and 485 nm [34].

### 2.8. PrestoBlue Viability Assay

For analysis of cell survival, HCASMCs (3 × 10^4^ cells/mL) were treated with or without 25 µg/mL oxLDL, PACAP38 (0.1 nM; 0.5 nM; 1.0 nM), and/or Maxadilan (0.1 nM; 0.5 nM; 1.0 nM) for 24 h. Cell viability was assessed using PrestoBlue™ (Invitrogen, Carlsbad, CA, USA) [38], according to the manufacturer’s protocol. Briefly, 1 h after the addition of PrestoBlue™, the optical density (OD) was measured at 570 nm and 600 nm (as a reference) using a SUNRISE ELISA reader (Tecan, Salzburg, Austria). Results are presented as % survival = [Sample OD (570 nm − 600 nm reference) × 100)/Control OD (570 nm − 600 nm reference)]. As control, (=100%) cells were cultured with medium alone (i.e., without addition of test substances) [38].

### 2.9. Propidium-Iodide-Staining

To investigate the effect on HCASMC apoptosis, a propidium iodide staining assay was performed. The HCASMCs were seeded onto a plate coated with extracellular matrix and incubated at 37 °C until the cells were 80% confluent. Subsequently, HCASMCs were treated with or without 25 µg/mL oxLDL, 0.5 nM PACAP, and/or 0.5 nM Maxadilan for 24 h at 37 °C. Cells were stained with PI (1:500, Invitrogen, Carlsbad, CA, USA) for 30 min at 37 °C. They were then gently washed once with PBS. PI-positive cells were imaged under a fluorescence microscope (Axiovert 135, Carl Zeiss AG, Oberkochen, Germany). The percentage of the PI-positive cell area relative to the total area was determined using ImageJ Fiji software.

### 2.10. Determination of Lipid Droplets (LDs) by BODIPY^TM^ 493/503

LDs are ubiquitous, dynamic organelles that serve as storage depots for neutral lipids, including triglycerides and cholesterol esters [39]. The fluorescent neutral lipid dye 4,4-difluoro-1,3,5,7,8-pentamethyl-4-bora-3a,4a-diaza-s-indacene (BODIPY™; Thermo Fisher Scientific, Waltham, MA, USA), which displays excitation (Ex)/emission (Em) maxima of 493/503 nm (Ex/Em = 493/503 nm), allows the quantification of the area containing neutral lipid. A total of 5% PFA-fixed HCASMCs were stained with 4 µM Bodipy^TM^493/503 (Thermo Fisher Scientific) and DAPI (1:1000) for 15 min and visualized using a Nikon Eclipse Ti laser-scanning microscope (Nikon GmbH, Düsseldorf, Germany). Cells were analyzed, and the fluorescence area was determined using ImageJ software (Fiji). Fluorometric measurements were performed at Ex/Em = 493/503 nm for BODIPY™ and at Ex/Em = 359/461 nm for DAPI using the Cytation3 microplate reader (BioTek Instruments Inc., Winooski, VT, USA). The Bodipy^TM^493/503 fluorescence dye values were normalized against DAPI-stained nuclei.

### 2.11. Scratch Assay

A scratch assay is a laboratory technique used to study cell migration and cell–cell interaction. HCASMCs were seeded in 24-well dishes and incubated at 37 °C for 24 h to establish a confluent monolayer. Scratches on the cell monolayer were made using a sterile 20 μL pipette tip (Time point: 0 h). After wounding, HCASMCs were treated with or without 25 µg/mL oxLDL, PACAP38 (0.5 nM), and/or Maxadilan (0.5 nM). Images were then taken at different time points (3 h, 4 h, 5 h, 24 h) using an inverted Axiovert 135 microscope, equipped with a motorized stage and a digital AxioCam MRc camera (Carl Zeiss AG, Oberkochen, Germany), to document wound closure. The images were analyzed with the MRI wound-healing tool (National Institutes of Health, Bethesda, MD, USA) using ImageJ software (Fiji). The wound closure rate was calculated by measuring the reduction in wound area over time.

### 2.12. Fluorescence Labeling of Mitochondria

To label the mitochondria, the HCASMCs were incubated with or without 25 µg/mL oxLDL, PACAP38 (0.5 nM), or Maxadilan (0.5 nM) in the presence of MitoTracker™ Green (1:1000, Molecular Probes, Eugene, OR, USA) for 24 h. After incubation, the cells were washed with phosphate-buffered saline (PBS) and visualized using a Nikon Eclipse Ti laser scanning microscope (Nikon GmbH, Düsseldorf, Germany) at Ex/Em = 488/523 nm. The images were analyzed using the Mitochondrial Network Analysis (MiNA) tool ImageJ software (Fiji) [40].

### 2.13. SDS-PAGE and Western Blot

After treatments with or without 25 µg/mL oxLDL, 0.5 nM PACAP38, or 0.5 nM Maxadilan, HCASMCs were washed with ice-cold PBS and lysed using radio-immunoprecipitation assay (RIPA) buffer pH 7.5 (Cell Signaling Technology, Frankfurt, Germany), supplemented with a protease/phosphatase inhibitor cocktail (Cell Signaling Technology). Protein concentrations were determined spectrophotometrically using the Pierce BCA (bicinchoninic acid) Protein Assay (Thermo Scientific, Rockford, IL, USA). Proteins were loaded onto NuPAGE^®^ Novex^®^ 4–12% Bis-Tris Gels, precast polyacrylamide gels (Life Technologies GmbH, Darmstadt, Germany). Proteins were transferred onto 0.45 μm nitrocellulose membranes (Millipore, Billerica, MA, USA) and stained with Ponceau S. Primary antibodies (Table S1) were added and incubated overnight at 4 °C in blocking buffer (5% fat-free milk). Membranes were incubated with enhanced ECL-anti-mouse IgG-POD antibody or ECL-anti-rabbit IgG-POD antibody (Table S1). The peroxidase reaction was visualized using the AceGlow chemiluminescence substrate (PEQLAB GmbH, Erlangen, Germany) and documented with the Fusion-SL Advance™ imaging system (PEQLAB GmbH, Erlangen, Germany), according to the manufacturer’s instructions. The intensities of the specific Western blot bands were quantified using Fiji ImageJ Gel-Analyzer (National Institutes of Health). The detected proteins were normalized to the total protein amount using Ponceau S staining.

### 2.14. ELISA

The intracellular level of human Ki67 was quantified using the DuoSet^®^ ELISA Development System (R&D Systems, Inc., Abingdon, UK). The Capture Antibody was coated onto a 96-well MaxiSorp-ELISA Microplate (Nunc, San Diego, CA, USA) and incubated overnight at room temperature. According to the manufacturer’s instructions, after the blocking step, the samples (2.5 μg protein/well) or standards were added to the wells. After incubation with the detection antibody and streptavidin–HRP, the substrate solution (SigmaFast™ OPD, Sigma-Aldrich Chemie GmbH) was added to each well and incubated for 30 min in the dark. The reaction was stopped with 50 μL of 3 M HCl. The Ki67 protein level (pg/mL) was measured with an ELISA reader (Tecan Deutschland GmbH, Crailsheim, Germany) at OD490/655 nm and normalized to crystal violet absorbance measured at 595 nm, with a reference at 660 nm.

### 2.15. Immunocytofluorescence Confocal Scanning Microscopy

Cells were fixed with ice-cold methanol and permeabilized with 0.1% Triton™X-100 in PBS. Thereafter, the detergent was removed by repeated washing in PBS. Primary antibodies (Cytochrom C (136F3), Cat. 4280, Cell Signaling Technology Inc., Danvers, MA, USA) were applied to PBS overnight (4 °C). After incubation with secondary antibodies (goat-anti-rabbit IgG H&L, F(ab’)2Fragment Alexa Fluor^®^ 488 Conjugate, Cat. #4412, Cell Signaling Technology Inc., Danvers, MA, USA) and subsequent staining with DAPI, the cells were covered with Immu-Mount™ (Thermo Electron Corporation; Pittsburgh, PA, USA). Images were taken with a confocal laser-scanning microscope, Eclipse Ti-E (Nikon GmbH, Düsseldorf, Germany), and analyzed using ImageJ Software (Fiji).

### 2.16. Statistical Analyses

Statistical analyses were performed using SigmaPlot 12 (Systat Software GmbH, Erkrath, Germany). After testing for normality (by Shapiro–Wilk), the unpaired Student’s *t*-test or one-way analysis of variance (ANOVA) was used. Data are reported as mean ± standard error of the mean (SD). *p* < 0.05 was considered statistically significant.

## 3. Results

### 3.1. PACAP Deficiency Affects Aortic Burden, Lumen Stenosis, and Body Weight in Mice

**Figure 1:**
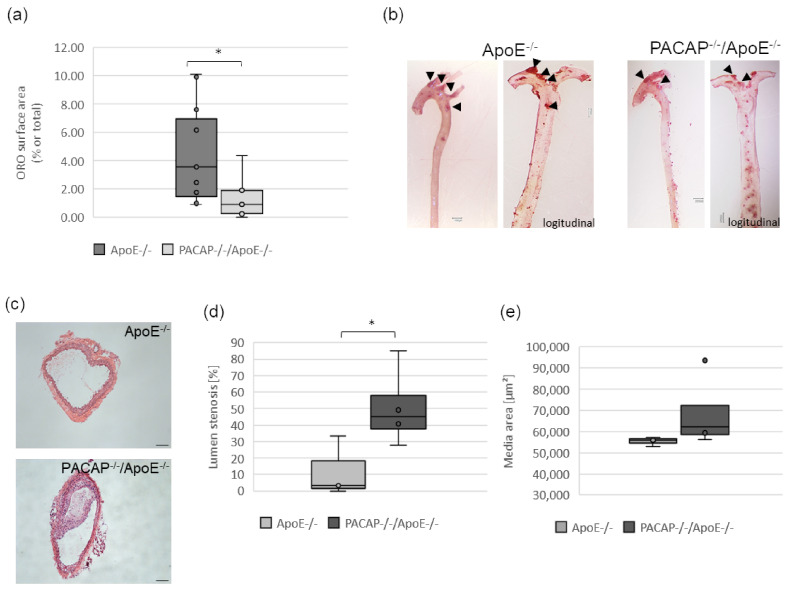
Effects of PACAP deficiency on aortic burden and lumen stenosis. (**a**) ORO-stained surface area (%) in ApoE^−/−^ (*n* = 15) and PACAP^−/−^/ApoE^−/−^ mice (*n* = 5) after 30 weeks of SC. (**b**) Representative images of the ORO-stained whole aorta and a longitudinal section of the aorta from ApoE^−/−^ and PACAP^−/−^/ApoE^−/−^ mice. Scale bar = 1000 µm. triangle, atherosclerotic plaque. (**c**) Representative HE-stained histological cross sections of BT from ApoE^−/−^ (*n* = 3) and PACAP^−/−^/ApoE^−/−^ mice (*n* = 5). Scale bar = 100 μm. (**d**) Lumen stenosis [%] and (**e**) media area [μm^2^] were measured in BT by computer-assisted morphometry. * *p* ≤ 0.05 vs. ApoE^−/−^ mice after 30 weeks of SC. BT, brachiocephalic trunc; SC, standard chow; *n*, number of experimental animals.

In this study, we demonstrated reduced aortic burden in PACAP^−/−^/ApoE^−/−^ male mice after 30 weeks on an SC compared to ApoE^−/−^ mice (*p* = 0.045) (Figure 1a,b). In addition, we demonstrated a 3.4-fold increase in luminal stenosis (*p* = 0.037) in PACAP^−/−^/ApoE^−/−^ mice after 30 weeks of SC in comparison with ApoE^−/−^ mice (Figure 1c,d), a finding that has also been observed in previous studies [21,22]. We also investigated the effects of PACAP deficiency on media thickness and body weight in ApoE^−/−^ mice: Media thickness in ApoE^−/−^ and PACAP^−/−^/ApoE^−/−^ mice at 30 weeks of SC remained unaffected (Figure 1e); body weight and BMI decreased after 30 weeks of SC in PACAP^−/−^/ApoE^−/−^ male mice compared to ApoE^−/−^ mice (Table 1).

### 3.2. Cell Migration of HCASMCs

The in vitro scratch assay is a well-known and widely used method for investigating cell migration and proliferation. This test is based on the observation that when the confluent HCASMCs layer is artificially scratched, the scratch closes as HCASMCs migrate toward the opening. By taking images during cell migration, we were able to quantify and analyze cell migration speed over time.

**Figure 2:**
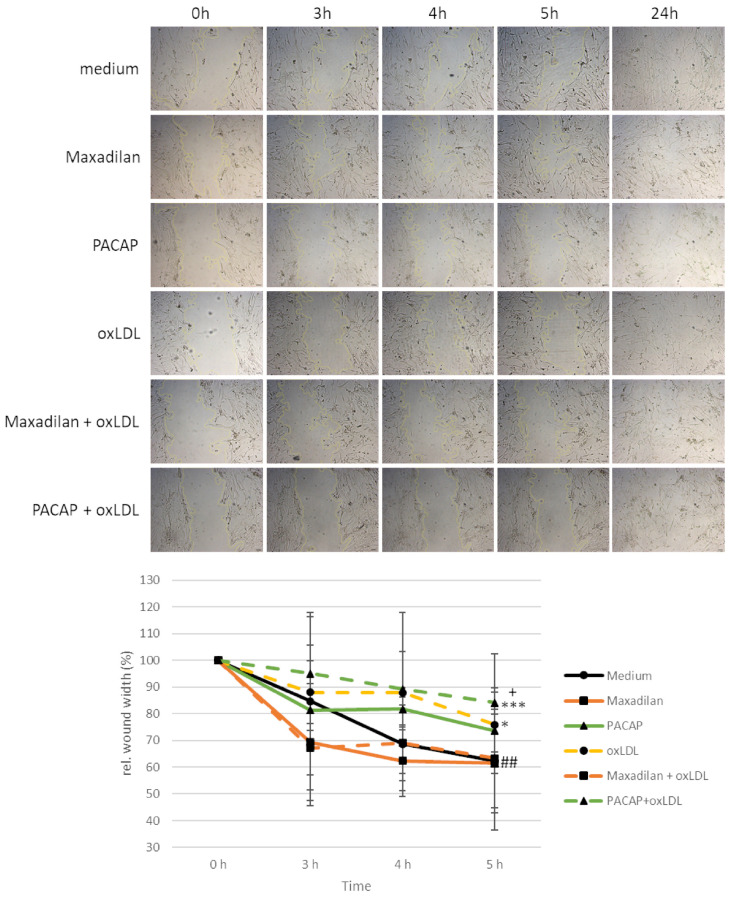
Effect of PACAP and Maxadilan on migration ability in cultured HCASMCs treated with 0.5 nM PACAP or 0.5 nM Maxadilan in combination with 25 µg/mL oxLDL or left untreated (medium). The migration was determined using a wound-healing assay. Bars represent means ± SEM of *n_scratch_* = 24 or *n_experiments_* = 8. Scala bar = 20 µm. *** *p* ≤ 0.001; * *p* ≤ 0.05 vs. medium; ^##^ *p* ≤ 0.01 vs. oxLDL; ^+^ *p* ≤ 0.05 vs. PACAP.

HCASMCs were able to close the scratch area by up to 38% under medium conditions after 5 h (Figure 2). After 24 h, the initial scratch area had completely closed in all treatment groups, indicating the complete reconstitution of the HCASMC monolayer (Figure 2). A total of 25 µg/mL oxLDL and 0.5 nM PACAP38 slowed HCASMC migration by up to 26% (*p* ≤ 0.001) and 16% (*p* ≤ 0.001), respectively, compared to the medium control after 5 h (Figure 2). Additionally, oxLDL inhibited the closure of the scratch area by 10% (*p* = 0.01) in PACAP38-treated HCASMCs compared to PACAP38 alone after 5 h (Figure 2). Maxadilan induced a faster closure of the scratch area in oxLDL-treated HCASMCs compared to oxLDL alone. Therefore, Maxadilan at a 0.5 nM concentration increased cell migration by 12% (*p* = 0.002) in HCASMCs compared to oxLDL-treated cells after 5 h (Figure 2).

### 3.3. Viability and Lipid Uptake in HCASMCs

**Figure 3:**
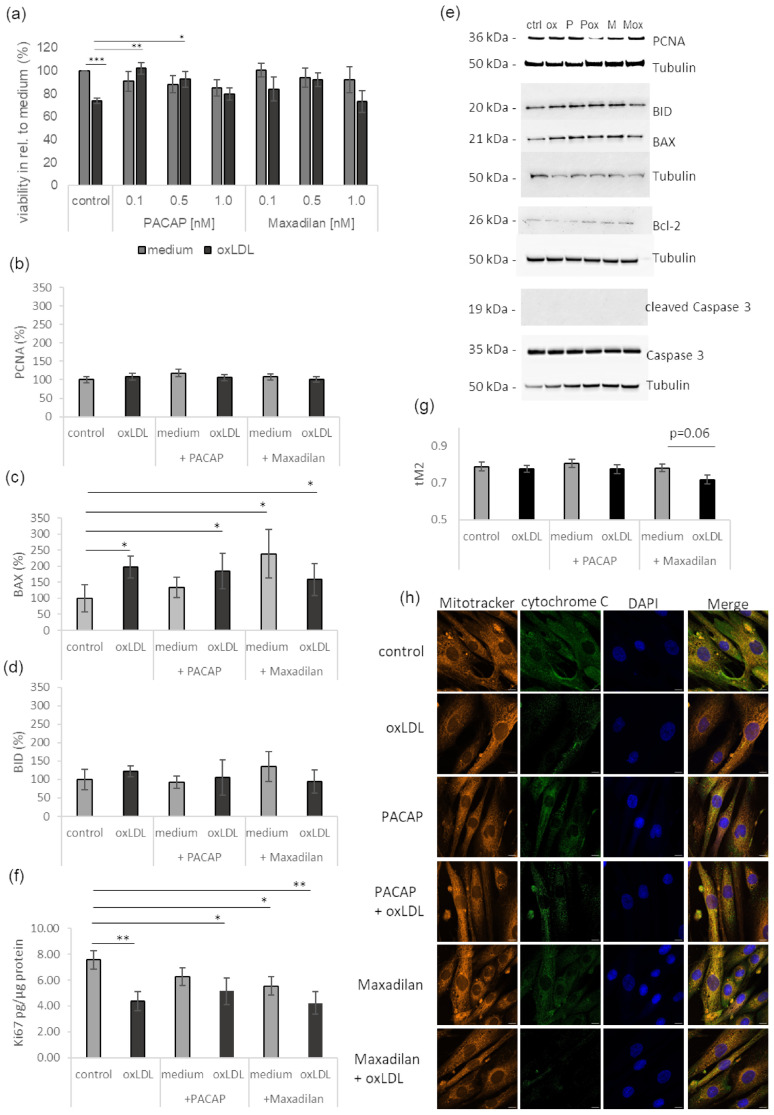
Analysis of apoptotic processes in HCASMCs treated with PACAP or Maxadilan in combination with 25 µg/mL oxLDL or left untreated (control). (**a**) The viability was determined by PrestoBlue^TM^ (*n* = 5). (**b**–**e**) Western blot analysis of (**b**) PCNA (*n* = 10), (**c**) BAX (*n* = 5), and (**d**) BID (*n* = 7) protein expression. (**e**) Representative western blot images. ctrl—control; ox—oxLDL; P—PACAP; M—Maxadilan; Pox—PACAP + oxLDL; Mox—Maxadilan + oxLDL. (**f**) ELISA analysis of Ki67 protein expression (*n* = 7). (**g**) Colocalization of Mitotracker Red with cytochrome C. The tM2 value indicates the overlap between the green fluorescence signal for cytochrome C and the red fluorescence signal for Mitotracker. n_images_ = 27 or n_experiments_ = 3. (**h**) Representative immunofluorescence images of mitochondria (red)/cytochrome C (green)/DAPI (blue) in HCASMCs, by confocal laser scanning microscopy (Nikon Eclipse). Scale bar = 10 µm; bars represent means ± SEM. *** *p* ≤ 0.001, ** *p* ≤ 0.01, * *p* ≤ 0.05 vs. control.

To monitor cell health, we used PrestoBlue HS cell viability reagents (Invitrogen). PACAP38 and Maxadilan alone showed no effect on the viability in HCASMCs compared to incubation with medium alone (Figure 3a). However, exposure to oxLDL resulted in a 26% decrease in viability (*p* ≤ 0.001), while PACAP38 significantly (*p* = 0.031) inhibited the oxLDL-induced decrease in viability by 28% and 19% at concentrations of 0.1 nM and 0.5 nM (Figure 3a).

In the present study, we investigated lipid uptake and viability in HCASMCs in relation to PACAP38 or Maxadilan. Due to its nonpolar structure, long-wavelength absorption, and fluorescence, Bodipy^TM^493/503 (Thermo Fisher Scientific, Waltham, MA, USA) was used as a dye to detect intracellular triglycerides (TAGs). ELISA measurements of Bodipy^TM^493/503 fluorescence showed that a 24 h oxLDL (25 µg/mL) incubation period resulted in increased BODIPY^TM^ fluorescence [normalized to DAPI fluorescence (Ex/Em = 359/457 nm)] in HCASMCs by 97% (*p* = 0.006) compared to the control, while PACAP38 and Maxadilan showed no significant effect on Bodipy^TM^493/503 fluorescence in HCASMCs when combined with oxLDL (Figure S1).

### 3.4. Apoptosis Analysis in HCASMCs

The Proliferating Cell Nuclear Antigen [PCNA] is an important cofactor of DNA synthesis and serves as a proliferation marker. Independent of the treatment of HCASMCs, its PCNA protein expression showed no significant changes (Figure 3b,e). BAX, a protein from the Bcl-2 family, can accelerate apoptosis processes. A total of 25 µg/mL oxLDL alone and combined with 0.5 nM PACAP or 0.5 nM Maxadilan increased BAX protein expression by 97% (*p* = 0.028), 85% (*p* = 0.05), and 59% (*p* = 0.029) compared to the control (Figure 3c,e and Figure S2). Additionally, 0.5 nM Maxadilan showed a significant increase of 133% (*p* = 0.011) in BAX protein expression (Figure 3c,e). Protein expression of BCL-2 was only detectably low in all samples (Figure 3e). Cleaved caspase-3 activates the proapoptotic protein BH3-interacting domain death agonist [BID], which is then converted into its active form and promotes the release of cytochrome C from the mitochondria to further drive apoptosis. Independent of the treatment of HCASMCs, cleaved caspase-3 and BID protein expression showed no significant changes (Figure 3d,e).

Ki67 is a protein that is present during the active G1, S, G2, and M phases of the cell cycle, while it is absent in the G0 phase of rested cells. A total of 25 µg/mL oxLDL alone and combined with 0.5 nM PACAP or 0.5 nM Maxadilan decreased the intracellular Ki67 concentration by 42% (*p* = 0.005), 32% (*p* = 0.031), and 44% (*p* = 0.002) compared to the control (Figure 3f). Additionally, 0.5 nM Maxadilan decreased the intracellular Ki67 concentration by 27% (*p* = 0.031) compared to the control (Figure 3f).

Additionally, we analyzed the co-localization of mitotracker red with cytochrome C (Figure 3g,h). Therefore, we used the Manders’ Colocalization Coefficient (MCC) [41]. The MCC is an image analysis method used to quantify the colocalization of two fluorescence signals. It measures the proportion of a protein that colocalizes with another, regardless of signal intensity. Values range from 0 (no overlap) to 1 (complete overlap). The tM2 value indicates the overlap between the green fluorescence signal and the red fluorescence signal. In general, there is a 79% overlap between cytochrome C signaling and Mitotracker Red in HCASMCs (Figure 3g). OxLDL reduced cytochrome C levels in the mitochondria of HCASMCs treated with Maxadilan compared to those treated with Maxadilan alone (Figure 3g,h).

### 3.5. Mitochondrial Morphology of HCASMCs

In our study, we used the Mitochondrial Network Analysis (MiNA) toolset, a pair of macros making use of existing ImageJ plugins, allowing for semiautomated analysis of mitochondrial morphologies in cultured mammalian cells [40,42]. MiNA groups rods, punctate, and single round structures together as individuals, while branched morphologies are categorized as networks [42]. This analysis allowed us to assess cell health, as previous studies have shown that fragmented mitochondria are the predominant morphology in mitochondrial dysfunction [43,44], while fused, network-like mitochondria are associated with cell survival mechanisms [45,46].

**Figure 4:**
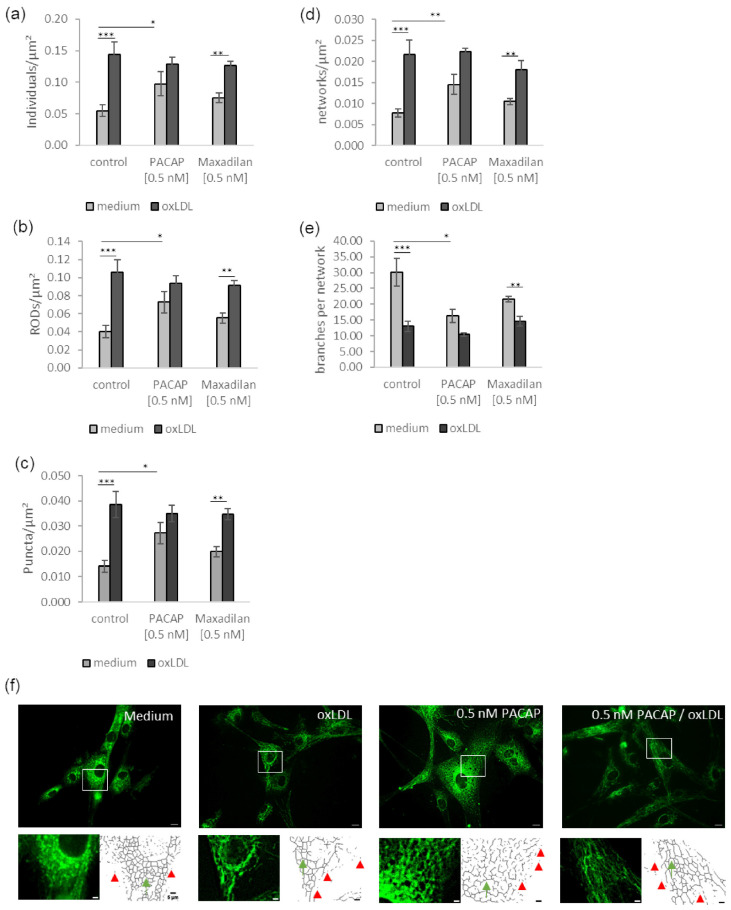
Mitochondrial morphology of HCASMCs with 0.5 nM PACAP or 0.5 nM Maxadilan in combination with 25 µg/mL oxLDL or left untreated (control). (**a**–**e**) MiNA descriptor analysis of mitochondrial morphology by detecting the number of (**a**) individuals, (**b**) RODs, (**c**) Puncta, (**d**) networks, and (**e**) mean network branch length. All data are representative of 12 images taken from 5 independent experiments. The bars represent means ± SEM. * *p* ≤ 0.05, ** *p* ≤ 0.001, *** *p* ≤ 0.0001 vs. medium. (**f**) Representative images of HCASMCs stained with Mitotracker green, a dye that localizes to actively respiring mitochondria. The white box shows the enlarged section. The black and white images are skeletonized images of HCASMCs. Red arrowhead, individuals; green arrow, networks. Scale bar = 5 µm.

Our results showed that 25 µg/mL oxLDL and 0.5 nM PACAP38 increased the number of individuals (red arrowhead) and the network structure (green arrow) and decreased the mean network branch length compared to medium control (Figure 4). Incubation of HCASMCs with oxLDL increased the number of RODs per µm^2^ area by 162% (*p* ≤ 0.001), the number of Puncta per µm^2^ area by 173% (*p* ≤ 0.001), as well as the individual structure per µm^2^ area by 165% (*p* ≤ 0.001) (Figure 4a–c). The network structure per µm^2^ area increased by 180% (*p* ≤ 0.001), whereas the mean network branch length decreased by 57% (*p* = 0.001) after 24 h oxLDL treatment (Figure 4d,e). A total of 0.5 nM PACAP38 increased the number of RODs per µm^2^ area by 80% (*p* = 0.028), the number of Puncta per µm^2^ area by 93% (*p* = 0.049), as well as the individual structure per µm^2^ area by 79% (*p* = 0.047) (Figure 4a–c). The network structure per µm^2^ area increased by 89% (*p* = 0.007), whereas the mean network branch length decreased by 46% (*p* = 0.016) after 24 h PACAP38 treatment (Figure 4d,e). Maxadilan showed no effects on the morphology of the mitochondrial structure (Figure 4a–e).

## 4. Discussion

VSMCs are present in all stages of atherosclerotic plaques and play a pivotal role in atherosclerotic development and progression. An abnormal proliferation of VSMCs promotes plaque formation and, in advanced plaque, prevents the rupture of the fibrous cap.

Previous studies have found that more than 80% of VSMCs in atherosclerotic plaques had lost the expression of contractile markers such as ACTA2. Furthermore, the SMC-specific Klf4 knockout model exhibited 50% smaller lesions and signs of increased plaque stability—including a doubling of the ACTA2-positive fibrin cap [9]. Thus, Klf4-dependent changes in the SMC phenotype and the effects appear to exacerbate the pathogenesis of the lesions. In contrast, the SMC-specific Oct4 knockout model showed an increase in lesion size and signs of reduced plaque stability, including the near-complete absence of an SMC-rich ACAT2-positive fibrin cap, reduced levels of mature collagen, increased lipid content, and increased intraplaque hemorrhage [11]. These findings suggest that cells derived from VSMCs in advanced atherosclerotic lesions in mice and humans exhibit far greater phenotypic plasticity than is generally assumed. Despite extensive research, the exact mechanisms involved in the development and progression of atherosclerotic lesions and the precise role of VSMCs remain largely unclear.

PACAP and its specific receptor PAC1 have already been detected in heart tissue and blood vessels [47,48,49], suggesting that PACAP signaling may play an important role in atherosclerosis. The clinical significance of atherosclerotic lesions depends on various characteristics, including lumen stenosis, plaque vulnerability, and plaque burden [50,51]. It has already been demonstrated in the pulmonary vasculature of wild-type mice that PACAP induces vasodilation in isolated pulmonary vessels and that the absence of its specific receptor, PAC1, after birth results in pulmonary hypertension and right heart failure. These in vivo findings demonstrate the critical importance of PAC1-mediated signaling for the maintenance of normal vascular tone [52]. In recent and previous studies, we showed that the deficiency of the neuropeptide PACAP resulted in increased lumen stenosis in the BT and aortic arch in ApoE^−/−^ mice fed with SC [21,22] and a decreased aortic plaque burden. PAC1 deficiency, on the other hand, results in a reduction in plaque development of atherosclerotic lesions in ApoE^−/−^ mice after 10 weeks or 20 weeks of cholesterol-enriched diet (CED) feeding [23]. ApoE^−/−^ mice treated with the PAC1 receptor agonist Maxadilan exhibit reduced atherosclerotic plaques after SC and CED, accompanied by reduced caspase-3 immunoreactive area in the tunica media, which is attributable to its anti-apoptotic action [25]. In this study, ApoE^−/−^ mice show plaque deposits primarily at the branching points leading to the BT, the left subclavian artery, and the left common carotid artery. In PACAP-deficient ApoE^−/−^ mice, plaque formation is concentrated primarily in the BT. Additionally, the composition of the plaque may also play a decisive role in this process. In earlier studies, plaque from PACAP-deficient ApoE^−/−^ mice showed higher levels of inflammation, increased collagen deposition, and increased necroptosis [21]. In cases of so-called negative remodeling, the vessel may constrict in response to chronic inflammation and the proliferation of connective tissue, thereby aggravating stenosis [53,54]. Also, the SCOT-HEART cohort study demonstrated that a high plaque burden predicts the risk of a heart attack—regardless of the severity of the luminal stenosis itself [55]. Another study by Mortensen et al. (2020) involving over 23,000 patients demonstrated that the risk of cardiovascular events increases linearly with the number of affected vascular segments (plaque burden)—even in patients who, according to angiography, did not have significant stenosis (less than 50%) [56].

In this study, we observed that PACAP significantly increased the viability reduced by oxLDL in HCASMCs, and Maxadilan tended to increase it as well. Takei et al. (2000) showed that PACAP regulates cell proliferation and differentiation, thereby promoting cell survival in basal forebrain cholinergic neurons [57]. The protective effect of PACAP has also been demonstrated in diabetes research and in traumatic injuries, as well as in sepsis-associated lung injuries [58,59,60].

Previous studies indicate that intimal VSMCs in advanced atherosclerotic lesions (types II and IV) express MΦ markers, have a selective reduction in ABCA1 expression, and develop into foam cells [14,61,62,63]. Additionally, in vitro data show that VIP and PACAP influenced the oxLDL-induced foam cell formation of cultured THP-1 MΦ or bone marrow-derived MΦ [21,22,64], probably via the VPAC1 receptor [22]. In this study, PACAP, as well as the specific PAC1 agonist Maxadilan [24,65,66], had no effects on the lipid accumulation in HCASMCs after oxLDL uptake.

The presence of a large number of intimal VSMCs implies that the migration of VSMCs from the media plays an important role during atherogenesis. Quantification of migration from the media to the intima in human vessels is not possible in vivo. Evidence indicates that human VSMCs can migrate in vitro in response to various stimuli, but the contribution of VSMC migration to the maturation of atherosclerotic plaques remains unclear. In this study, we used a wound-healing assay to analyze HCASMC migration in response to oxLDL, PACAP, and Maxadilan. Since we observed a change in wound closure within the first 5 h, we conclude that this is due to the cells’ migratory behavior, as observed by others [67]. We showed that Maxadilan improves HCASMC migration, which had been impaired by oxLDL. Maxadilan is a specific agonist of the PAC1 receptor, one of the receptors for PACAP, e.g., VPAC1-R and VPAC2-R. The PAC1 receptor is a class B G-protein-coupled receptor (GPCR). It is unique in that it is dual-coupled. This means that when its ligand PACAP binds to it, it simultaneously activates two major signaling cascades, with the activation of protein kinase C (PKC) playing a key role. In certain cancer cells, such as neuroblastoma cells, the activation of the PAC1 receptor stimulates cell migration. While cell division is regulated via the cAMP pathway, cell migration relies heavily on Gαq- and PKC-dependent regulation of the cytoskeleton (via the RhoA-ROCK signaling pathway) [68]. Furthermore, PACAP and oxLDL led to the impaired migration of HCASMCs. It is known that apolipoproteins retained in the intima undergo modifications to form oxLDL, which has been found to increase inflammation, unregulated LDL-C uptake, and VSMC proliferation, migration, and phenotypic switching [69,70,71,72]. Liu et al. (2014) demonstrated that oxLDL significantly promoted the proliferation and migration of HCASMCs in a dose-dependent manner using the Transwell assay by upregulating MMP-9 mRNA and protein levels [72]. OxLDL has also been shown to be cytotoxic and promote the apoptosis of cultured VSMCs [73,74,75,76], which we are able to verify using the viability assay. However, intimal regions of advanced plaques positive for oxLDL show the lowest smooth muscle actin immunoreactivity in the intima, which would be accompanied by reduced migration of VSMCs into the intima [77], and could confirm our in vitro data of oxLDL-induced slowed migration in wound-healing assays. We are not entirely clear whether human migration occurs independently or is dependent upon cell proliferation. It is known that intimal and medial regions of advanced plaques, where oxLDL is localized, show the highest BAX expression in media and intima [77]; these observations can also be confirmed by our in vitro data showing that oxLDL increases BAX expression in HCASMCs. The study by Kataoka et al. (2001) showed that oxLDL-induced BAX expression is dependent on lectin-like oxLDL receptor-1 (LOX-1) [76]. Bcl-2 family members have pro- and anti-apoptotic effects. Bcl-2 is an anti-apoptotic protein, while Bad and BAX are pro-apoptotic. In our study, the protein expression of BCL-2 was only detectably low in all samples. Also, we show that PACAP did not affect BAX expression, suggesting that decreased migration by PACAP is not only influenced by cell death. It is known that cAMP, which activates PKA, can inhibit the migration of SMCs following injury [78,79]. PKA is activated by the binding of PACAP to its Gαs-coupled receptors (VPAC1/2, PAC1) and the resulting increase in cAMP concentration [17]. Interestingly, Maxadilan shows reduced Ki67 protein levels, a marker of cell proliferation, with increased BAX levels accompanied by increased oxLDL-induced migration inhibition. Using propidium iodide staining (Figure S3), cytochrome C staining, and cleaved caspase-3 Western blot analysis, we were unable to detect any evidence of apoptotic processes despite increased BAX expression and reduced Ki67 protein levels in Maxadilan-treated HCASMCs. Elevated BAX expression without cleaved caspase-3 and cytochrome C release suggests that the cell initiates apoptosis but does not complete it. This may be due to inhibitory mechanisms in the caspase cascade. Further detailed analyses of the apoptotic signaling pathway (e.g., caspase 9, IAPs) are necessary to identify the exact signaling pathways. In summary, this study did not fully clarify the extent to which PACAP or Maxadilan influences proliferation and apoptosis processes in HCASMCs upon oxLDL incubation.

Mitochondria are highly dynamic organelles. Several studies indicate that mitochondrial dynamics play crucial roles in cell viability, intracellular signaling, aging, mitochondrial health, as well as their bioenergetic function and quality control [80,81]. Like other cellular endomembranes, they continuously move along the cytoskeleton and regularly interact through membrane anchoring, fusion, and fission. These fusion and fission processes can reveal mitochondrial quality. Normally, a balance between fission and fusion maintains mitochondrial function [42].

Mitochondrial dysfunction inhibits fusion and ongoing fission processes, which separate non-functional mitochondria from the healthy mitochondrial network [82]. The dysfunctional mitochondria are then degraded by mitophagy [82]. Mitochondrial fusion serves as a survival mechanism by mixing mitochondrial contents and enabling the replenishment of depleted cellular resources, such as lipids and proteins, thereby compensating for functional deficits within the mitochondrial network [82]. Stress conditions can also induce fusion (stress-induced mitochondrial hyperfusion; SIMH), which leads to the elongation of the mitochondrial tubules and protection from mitophagy [82]. Therefore, recent studies provide evidence that mitochondrial dynamics play an important role in various diseases, such as CVDs and neurodegenerative diseases [83,84,85,86]. In this study, we used the MiNA tool [42], an ImageJ macro, to analyze the mitochondrial structure of fluorescently labeled cell lines. In a fragmented state, mitochondria are more spherical, rounded individuals, while hyperfused mitochondria morph into large networks with a longer network branch length [42]. At first, we show a significant increase in fragmented mitochondria by an increased number of individuals per µm^2^ (Puncta, RODs) and an increase in smaller networks per µm^2^ with a shorter mean branch length in oxLDL and PACAP-treated HCASMCs compared to medium (control). The total number of mitochondria, i.e., the sum of the number of individuals and networks, therefore also increased. Previous studies suggest that mitochondrial fragmentation is beneficial for uncoupled respiration [81,87]. Increased decoupled respiration leads to reduced bioenergetic efficiency, meaning that energy obtained from nutrient oxidation is diverted to heat production. Reduced bioenergetic efficiency can serve as a protective mechanism against the harmful effects of nutrient overload, including the associated increased removal of excess nutrients and their potentially cytotoxic metabolites [81]. Several studies have shown that oxLDL induces cell death in cells of the annulus fibrosus [88] and vascular endothelial cells [89] by regulating the DRP1-mediated mitochondrial fission pathway. Furthermore, PACAP induced the fission pathway in the mitochondria of HCASMCs with increased viability. Healthy cells are able to rapidly remodel their mitochondria to maintain energy homeostasis. The fission and fusion of mitochondria can therefore also be modulated as a compensatory mechanism to maintain the pool of healthy mitochondria in cells [90,91,92,93]. However, the data we have presented primarily describe morphological changes in the mitochondria rather than functional changes. Therefore, further analyses will reveal the extent to which PACAP/Maxadilan induces functional changes in the mitochondria.

Our results illustrate the ambivalence of the PACAP/PAC1 signaling pathway in atherogenesis. Current in vitro data show the direct cell-protective effects (viability) of PACAP and complex control of migration via the PAC1 agonist Maxadilan. Furthermore, our findings suggest that the regulation of mitochondrial dynamics is a key mechanism through which PACAP modulates the cellular response of VSMCs to atherogenic stress factors. Future studies will need to clarify the underlying molecular mechanisms of this stress-induced mitochondrial remodeling and its relevance for the phenotypic plasticity of VSMCs in the context of atherosclerosis in order to fully evaluate the therapeutic potential of the PACAP/PAC1 system.

## Figures and Tables

**Table 1:** Effects of PACAP^−/−^ on body weight, body size, and BMI.

	Age [Weeks]	Body Weight [g]	Body Height [cm]	BMI [g/cm^2^]	*n*
ApoE^−/−^	31.68 ± 3.47	30.08 ± 2.49	9.39 ± 0.48	3.21 ± 0.29	19
PACAP^−/−^/ApoE^−/−^	30.23 ± 1.86	27.17 *** ± 1.67	9.19 ± 0.67	2.97 ** ± 0.22	11
*p* value	1.00	≤0.001	0.18	≤0.01	

** *p* ≤ 0.01; *** *p* ≤ 0.001 vs. ApoE^−/−^; mean ± SD; PACAP, pituitary adenylate cyclase-activating polypeptide; BMI, body mass index.

## Data Availability

Data and materials are available on request.

## Supplementary Materials

The following supporting information can be downloaded at: https://www.mdpi.com/article/10.3390/cells15121127/s1, Table S1. List of antibodies for western blot. Figure S1. Effect of exogenous PACAP38 (PACAP) or Maxadilan on lipid storage in cultured HCASMCs treated (24 h) with 0.1 nM, 0.5 nM, or 1.0 nM PACAP38 or Maxadilan, in combination with 25 µg/ml oxLDL or left untreated (control). Intracellular lipid droplets were detected using BODIPYTM 493/503. Bars represent means ± SEM of 4 experiments. Scala bar = 50 µm. ** *p* ≤ 0.01, * *p* ≤ 0.05 vs. control. Figure S2. Western blot images of BAX and Ponceau staining of HCASMCs treated with PACAP [0.5 nM] or Maxadilan [0.5 nM] in combination with 25 µg/ml oxLDL or left untreated (control). ctrl—control; ox—oxLDL; P—PACAP; M—Maxadilan; Pox—PACAP + oxLDL; Mox—Maxadilan + oxLDL. Figure S3. Analysis of apoptotic processes in HCASMCs treated with 0.5 nM PACAP or 0.5 nM Maxadilan in combination with 25 μg/ml oxLDL, or untreated (control). Apoptosis was detected using propidium iodide (PI; Invitrogen Life technologies Corporation, Eugene, Oregon, USA) staining according to the manufacturer’s protocol and visualized using an Axiovert 135 inverted microscope and an AxioCam MRc digital camera (Carl Zeiss AG, Oberkochen, Germany). The cells were analyzed, and the fluorescence area [%] was determined using ImageJ 1.54p (Fiji) software.

## Abbreviations

The following abbreviations are used in this manuscript:

ACTA2	Alpha-actin-2
BID	BH3-interacting domain death agonist
BMI	Body mass index
BT	Brachiocephalic trunk
cAMP	Cyclic adenosine monophosphate
CED	Cholesterol-enriched diet
CVDs	Cardiovascular diseases
ECM	Extracellular matrix
GPCR	G-protein-coupled receptor
HCASMCs	Human coronary artery smooth muscle cells
HE	Hematoxylin–eosin
Klf4	Krüppel-like factor 4
LD	Lipid droplet
LDL	Low-density lipoprotein
LOX-1	Lectin-like oxLDL receptor-1
MՓs	Macrophages
MCC	Manders’ Colocalization Coefficient
MiNA	Mitochondrial Network Analysis
nLDL	Native LDL
Oct4	Octamer-binding protein 4
ORO	OilRedO
oxLDL	Oxidized LDL
PACAP	Pituitary adenylate cyclase-activating polypeptide
PI	Propidium odide
PKA	Protein kinase A
PKC	Protein kinase C
PCNA	Proliferating Cell Nuclear Antigen
PCR	Polymerase chain reaction
REM	Relative electrophoretic mobility
RT	Room temperature
SC	Standard chow
SIMH	Stress-induced mitochondrial hyperfusion
SMCs	Smooth muscle cells
TAGs	Triglycerides
TNBSA	2,4,6-trinitrobenzene sulfonic acid
VSMC	Vascular SMC
