# Supplementary material for: PACAP and Maxadilan (PAC1 Agonist) Influence Plaque Progression, Migratory Ability, and Mitochondrial Morphology and Dynamics in Vascular Smooth Muscle Cells
Source: Cells. 2026 Jun 22;15(12):1127. doi: 10.3390/cells15121127 (PMC13296632; doi:10.3390/cells15121127)
Supplement: Supplementary file 1 [file cells-15-01127-s001.zip › Table S1.pdf]

Table S1. List of antibodies for western blot.

Name	Catalog number	Company	Dilution
**Primary Antibody**			
**Anti-PCNA (D3H8P)**	#13110	Cell Signaling Technology Inc., Danvers, USA	1:1000
**Anti-Bax [E63]**	ab32503	Abcam plc., Cambridge, UK	1:1000
**Anti-BID**	PA5-29159	Invitrogen Thermo Fisher Scientific, Massachuates, USA	1:1000
**Anti-Caspase 3**	Ab44976	Abcam plc., Cambridge, UK	1:1000
**Anti-cleaved Caspase 3 (Asp175) (5A1E)**	#9664	Cell Signaling Technology Inc., Danvers, USA	1:200
**Anti-Bcl2**	PAS-27094	Invitrogen Thermo Fisher Scientific, Massachuates, USA	1:500
**Anti-α-Tubulin**	Ab4074	Abcam plc., Cambridge, UK	1:6000
**Secondary Antibody**			
**ECL-anti-rabbit IgG-POD**	NA9340	GE Healthcare Life Science Freiburg	1:3000
**ECL-anti-mouse IgG-POD**	NA9310	GE Healthcare Life Science Freiburg	1:3000
