# Supplementary material for: PACAP and Maxadilan (PAC1 Agonist) Influence Plaque Progression, Migratory Ability, and Mitochondrial Morphology and Dynamics in Vascular Smooth Muscle Cells
Source: Cells. 2026 Jun 22;15(12):1127. doi: 10.3390/cells15121127 (PMC13296632; doi:10.3390/cells15121127)
Supplement: Supplementary file 1 [file cells-15-01127-s001.zip › Figure S1.pdf]

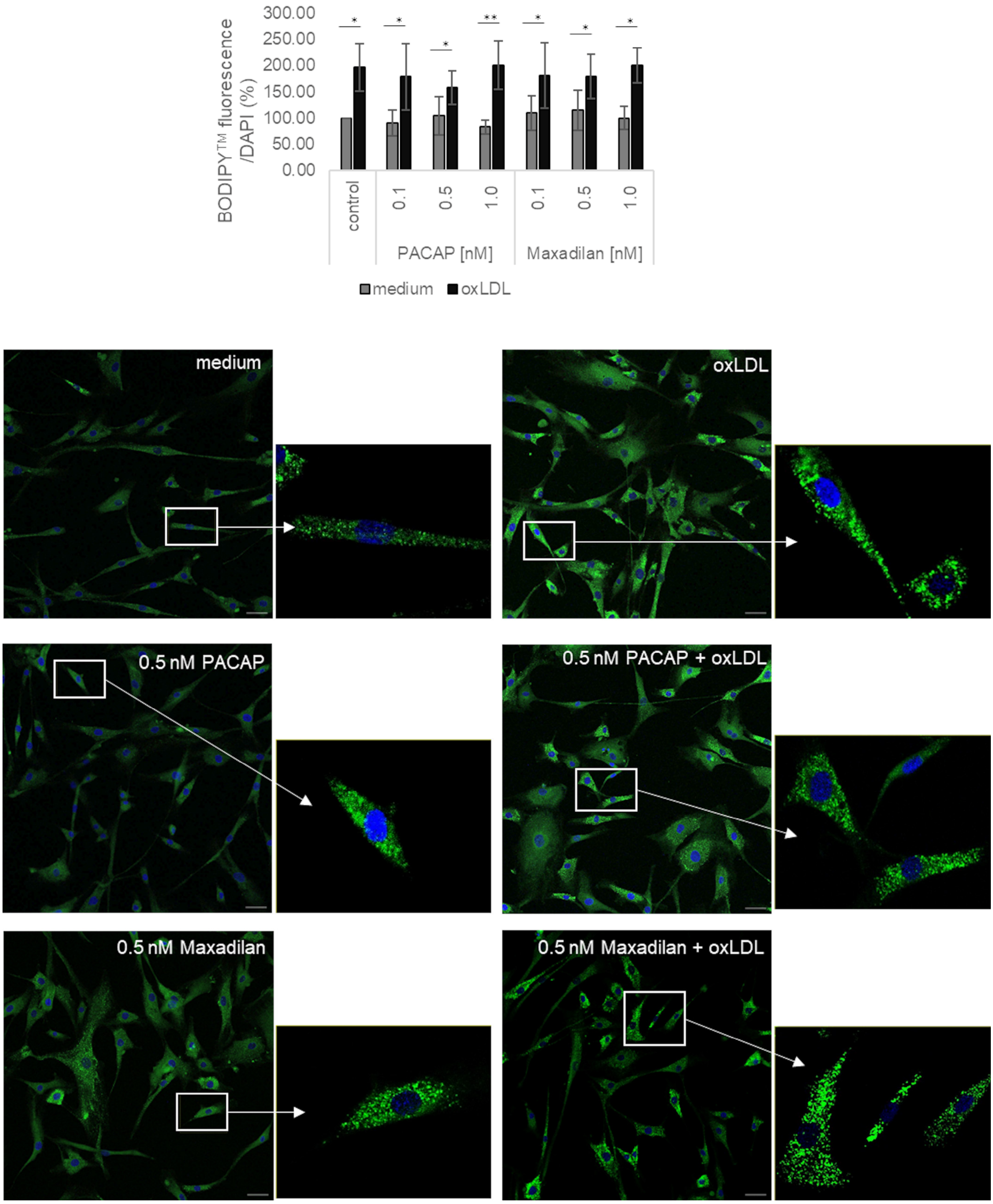

Figure S1. Effect of exogenous PACAP38 (PACAP) or Maxadilan on lipid storage in cultured HCASMCs treated (24h) with 0.1 nM, 0.5 nM, or 1.0 nM PACAP38 or Maxadilan, in combination with 25 µg/ml oxLDL or left untreated (control). Intracellular lipid droplets were detected using BODIPY™ 493/503. Bars represent means ± SEM of 4 experiments. Scala bar = 50 µm. $^{**}p \leq 0.01$, $^{*}p \leq 0.05$ vs. control.
