# Supplementary material for: PACAP and Maxadilan (PAC1 Agonist) Influence Plaque Progression, Migratory Ability, and Mitochondrial Morphology and Dynamics in Vascular Smooth Muscle Cells
Source: Cells. 2026 Jun 22;15(12):1127. doi: 10.3390/cells15121127 (PMC13296632; doi:10.3390/cells15121127)
Supplement: Supplementary file 1 [file cells-15-01127-s001.zip › Figure S2.pdf]

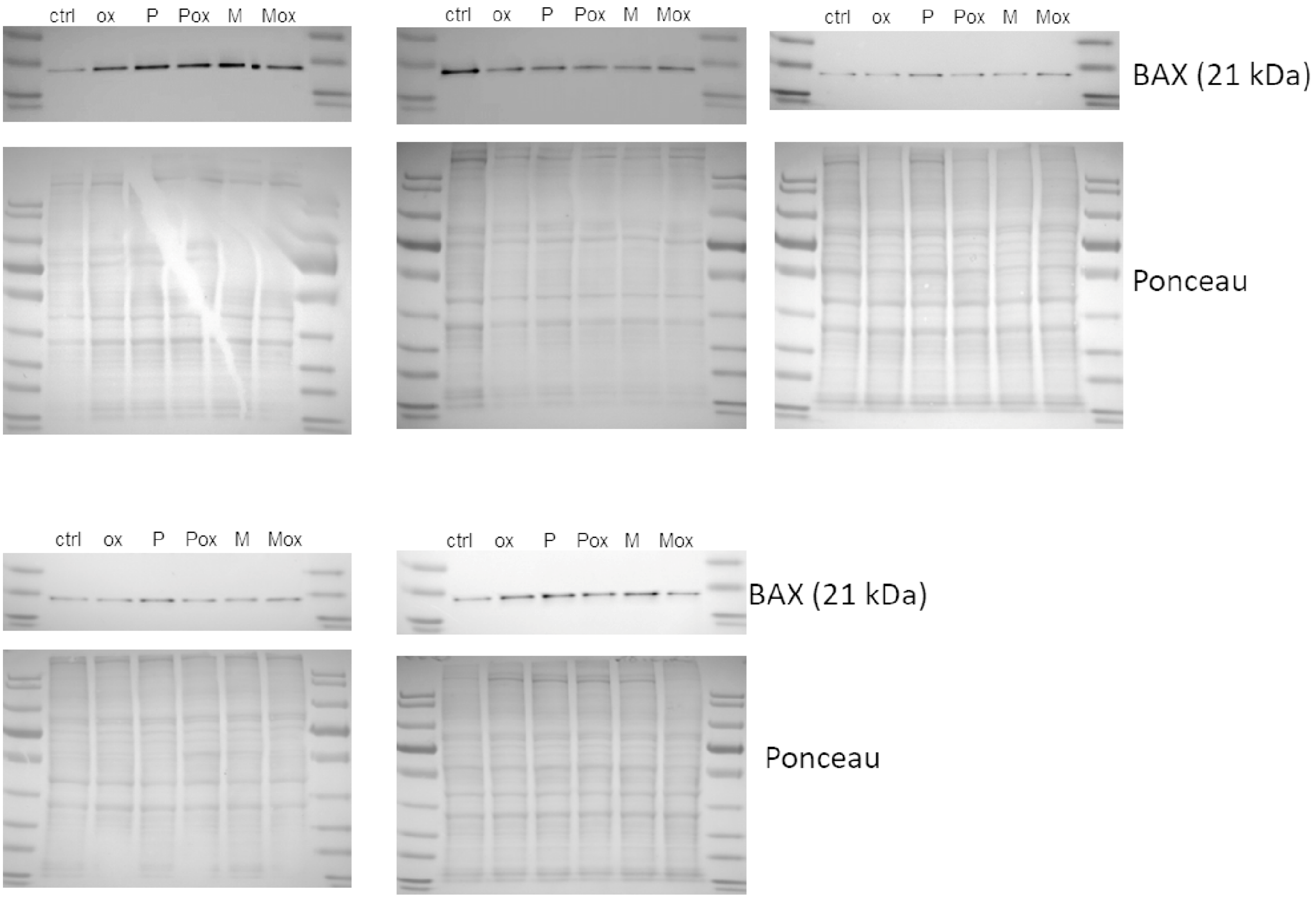


**Figure S2.** Western blot images of BAX and Ponceau staining of HCASMCs treated with PACAP [0.5 nM] or Maxadilan [0.5 nM] in combination with 25 μg/ml oxLDL or left untreated (control). ctrl - control; ox - oxLDL; P - PACAP; M - Maxadilan; Pox - PACAP + oxLDL; Mox - Maxadilan + oxLDL.
