# Supplementary material for: PACAP and Maxadilan (PAC1 Agonist) Influence Plaque Progression, Migratory Ability, and Mitochondrial Morphology and Dynamics in Vascular Smooth Muscle Cells
Source: Cells. 2026 Jun 22;15(12):1127. doi: 10.3390/cells15121127 (PMC13296632; doi:10.3390/cells15121127)
Supplement: Supplementary file 1 [file cells-15-01127-s001.zip › Figure S3.pdf]

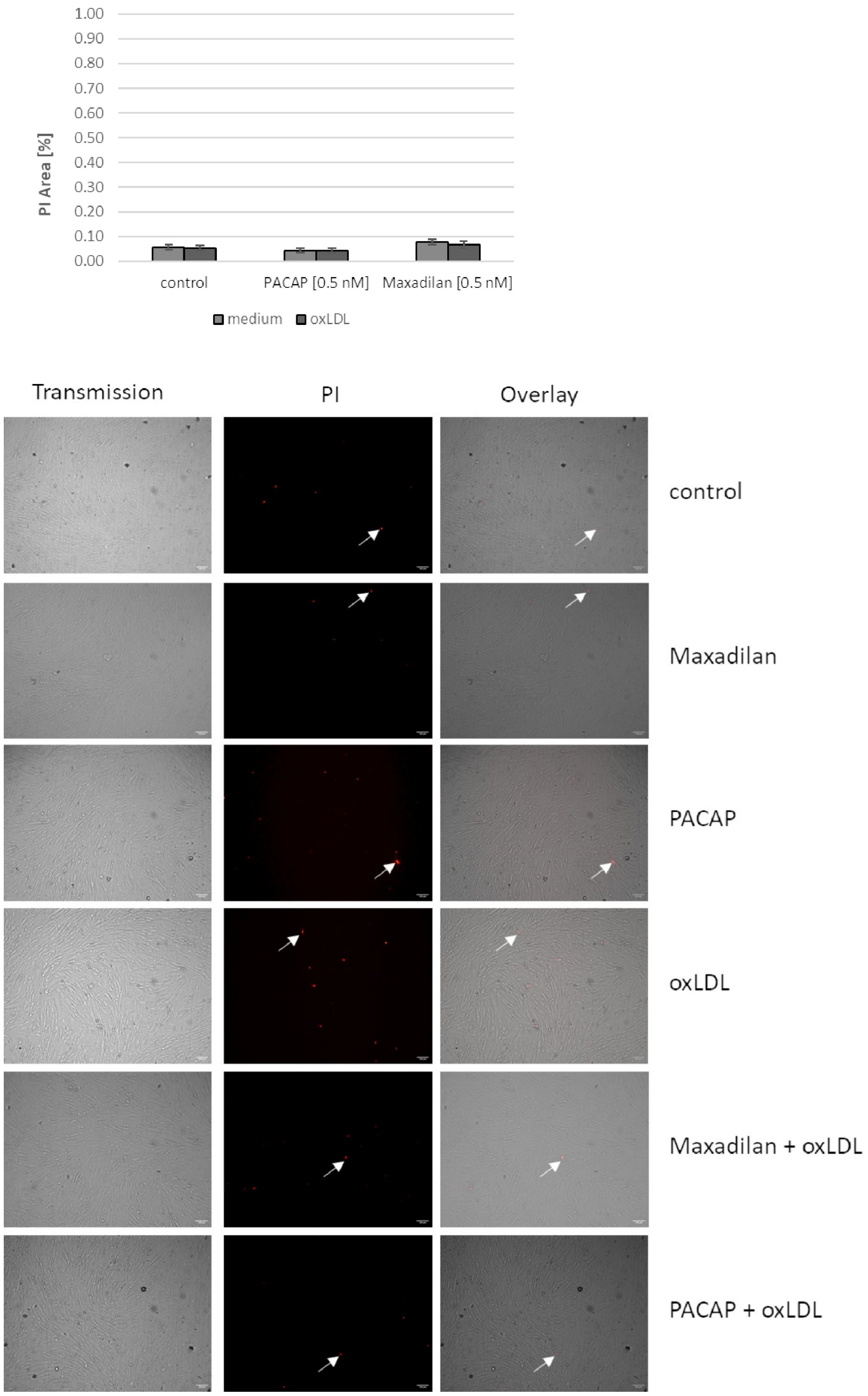

Figure S3. Analysis of apoptotic processes in HCASMCs treated with 0.5 nM PACAP or 0.5 nM Maxadilan in combination with 25 µg/ml oxLDL, or untreated (control). Apoptosis was detected using propidium iodide (PI; Invitrogen Life technologies Corporation, Eugene, Oregon, USA) staining according to the manufacturer's protocol and visualized using an Axiovert 135 inverted microscope and an AxioCam MRc digital camera (Carl Zeiss AG, Oberkochen, Germany). The cells were analyzed, and the fluorescence area [%] was determined using ImageJ 1.54p (Fiji) software.
